# Metastasis patterns and prognosis in young breast cancer patients: A SEER database analysis

**DOI:** 10.3389/fonc.2022.872862

**Published:** 2022-10-13

**Authors:** Weifang Zhang, Shang Wu, Jinzhao Liu, Xiangmei Zhang, Xindi Ma, Chao Yang, Miao Cao, Shuo Zhang, Yunjiang Liu

**Affiliations:** ^1^ Breast Cancer Center, The Fourth Hospital, Hebei Medical University, Shijiazhuang, China; ^2^ The Second Department of Thyroid Breast Surgery, Cangzhou Central Hospital, Cangzhou, China; ^3^ Research Center, The Fourth Hospital, Hebei Medical University, Shijiazhuang, China

**Keywords:** breast cancer, young patients, metastasis patterns, SEER, prognosis

## Abstract

**Background:**

There are few studies on young patients with metastatic breast cancer (MBC). This study aims to explore the metastasis pattern and prognosis of young patients with MBC.

**Methods:**

A total of 6,336 MBC patients diagnosed in the Surveillance, Epidemiology, and End Results (SEER) database from 2010 to 2015 were selected. They were divided into two age groups: the younger group (≤40 years old) and the older group (>40 years old). χ^2^ test was used to compare clinicopathological characteristics. Survival differences were compared by Kaplan–Meier analysis. Cox regression models were used to determine the prognostic factors affecting survival. Propensity score matching (PSM) was performed to balance the effects of baseline clinicopathological differences.

**Results:**

Finally, 494 patients (7.8%) who are ≤40 years old and 5,842 patients (92.2%) who are >40 years old were included. In the younger group, the proportion of liver metastasis was significantly higher than that in the older group; the proportion of lung metastasis was significantly lower than that of the older group. Kaplan–Meier analysis showed that the younger group had the best prognosis and the older group had the worst. Youth is an independent protective factor for overall survival (OS). In the younger group, liver metastasis had the best prognosis among all metastatic sites, and the HER2-enriched subtype had the best prognosis among all subtypes.

**Conclusions:**

The disease in young MBC patients is more aggressive but has a better prognosis, especially in liver metastases and the HER2-enriched subtypes.

## Introduction

Breast cancer (BC) is the most common cancer in women and the leading cause of cancer-related deaths among women worldwide (1). For breast cancer, “young patients” are women diagnosed with cancer before age 40 (2). Breast cancer in women less than 40 years of age accounts for about 7% of breast cancers, making it the most common cancer diagnosed in women for the 25–39 years old age group (3) (4). The number of patients with metastatic breast cancer under 40 years old showed a trend of stable and even accelerated growth (5). Compared with developed countries, breast cancer in young women is a huge burden in developing countries. A disproportionate number of young women lose their lives every year because of this type of cancer (6). Moreover, young women are usually at the peak of their careers when they have to deal with a sudden diagnosis of cancer. Juggling a job, the pressures of being young parents, if relevant, concerns with preexisting financial issues, and peer pressures concurrently with a cancer diagnosis can affect adherence to therapies (7). Therefore, in-depth research on breast cancer in young women is necessary.

In metastatic breast cancer (MBC), the most common sites of metastasis include the bone, brain, lung, and liver (8). Currently, short metastasis-free interval, visceral involvement and crisis, negative hormone receptor and particularly triple-negative subtype, primary endocrine resistance for luminal subtype, and a number of metastatic sites are recognized as poor prognostic factors (9). However, the effect of age on prognosis remains unclear. Studies have shown that age at diagnosis is associated with breast cancer survival, but the results are conflicting. Some studies (10, 11) have shown that youth is associated with poor prognosis because tumors are more aggressive in young people. Conversely, other studies have shown that older patients have a worse prognosis than younger patients (12–14). Therefore, the purpose of this study is to explore the metastasis pattern and prognosis of MBC patients aged ≤40 years.

## Material and methods

### Data collection

Data were obtained from the US Surveillance, Epidemiology, and End Results (SEER) with the username 10067-Nov2018. The data we selected came from Incidence-SEER Research Plus Data, 9 Registries, based on the November 2020 submission. Since this study used registry data, this study was exempted by the ethics committee of the Forth Hospital of Hebei Medical University. The methods were based on approved guidelines (15).

### Study population

Patients diagnosed with advanced breast cancer from 1 January 2010 to 31 December 2015 were included in the study. The inclusion criteria were as follows: 1) female patients, 2) primary site at the breast, 3) diagnosis between 2010 and 2015, and 4) stage IV of the 7th edition of the American Joint Committee on Cancer (AJCC) staging system. The exclusion criteria were as follows: 1) reported only from a nursing or convalescent home, hospice, autopsy, or death certificate, and 2) patients with an unknown metastatic site. Patients were divided into two groups according to age (younger group, ≤40 years old; older group, >40 years old). A total of 6,337 patients were included. The following factors were extracted: age of diagnosis, year of diagnosis, race, TNM stage (AJCC 7th edition), BC subtype, surgery, chemotherapy, site of distant metastasis (bone, brain, liver, lung, and distant lymph nodes), death events, and survival time.

### Statistical analysis

Overall survival (OS) was calculated from the date of diagnosis to the date of death due to any cause, the date of the last follow-up, or 31 December 2015. Breast cancer-specific survival (BCSS) was measured as the time from the date of diagnosis to the date of death attributed to breast cancer. The clinical characteristics of the selected patients were compared with Pearson’s χ^2^ test. Survival was estimated using the Kaplan–Meier method and compared between the different metastatic groups using a log-rank test. The survival curves were drawn with GraphPad Prism 7.0.0. Hazard ratios (HRs) and 95% confidence intervals (CIs) were calculated by univariable and multivariable Cox regression models to assess the effect of the factors associated with OS and BCSS. Propensity score matching (PSM) was performed to balance the effects of baseline clinicopathological differences. All statistical analyses were performed using SPSS Statistics 25.0. Statistical significance was considered at a two-sided p-value <0.05.

## Results

### Demographics

Overall, 6,336 MBC patients were enrolled in our study from the SEER database. Clinical characteristics of patients in two age groups are summarized in **Table 1**. A total of 494 patients (7.8%) were diagnosed at the age of ≤40 years and 5,842 patients (92.2%) at >40 years. There were significant differences among the two groups in race, T stage, N stage, BC subtype, surgery, chemotherapy, and metastatic sites. In the younger group, there were more Black people than any other race (25.9% vs. 16.6%, p < 0.001) and fewer White people (59.7% vs. 74.4%, p < 0.001). The younger group had larger tumor size with more stage T2/T3 (51.8% vs. 41.0% and 11.8%, p < 0.001) tumors, as well as a higher rate of lymph node involvement (p < 0.001). The younger group was more likely to have the more aggressive subtypes—Luminal B, HER2-enriched, and triple-negative subtypes (p < 0.001), while the older group was more likely to have the luminal A subtypes. In terms of treatment, the younger group had a higher rate of receiving surgery (41.3% vs. 28.1%, p < 0.001) and chemotherapy (81.2% vs. 51.3%, p < 0.001).

**Table 1:** Comparison of the clinical and pathological characteristics between two age groups before and after PSM.

	Before PSM			After PSM		
	≤40 years old	>40 years old	*p*	≤40 years old	>40 years old	*p*
Variable	N = 494	N = 5,842		N = 427	N = 427	
Race			<0.001			0.488
White	295 (59.7%)	4,348 (74.4%)		280 (65.6%)	272 (63.7%)	
Black	128 (25.9%)	967 (16.6%)		105 (24.6%)	102 (23.9%)	
Other^a^/unknown	71 (14.4%)	527 (9.02%)		42 (9.84%)	53 (12.4%)	
T stage			<0.001			0.739
T0/T1	57 (11.5%)	846 (14.5%)		43 (10.1%)	53 (12.4%)	
T2/T3	256 (51.8%)	2,396 (41.0%)		225 (52.7%)	223 (52.2%)	
T4	127 (25.7%)	1,710 (29.3%)		110 (25.8%)	104 (24.4%)	
Tx	54 (10.9%)	890 (15.2%)		49 (11.5%)	47 (11.0%)	
N stage			<0.001			0.322
N0	83 (16.8%)	1,436 (24.6%)		68 (15.9%)	79 (18.5%)	
N1/N2	291 (58.9%)	2,985 (51.1%)		263 (61.6%)	254 (59.5%)	
N3	99 (20.0%)	823 (14.1%)		76 (17.8%)	65 (15.2%)	
Nx	21 (4.25%)	598 (10.2%)		20 (4.68%)	29 (6.79%)	
Radiation			<0.001			0.146
No	265 (53.6%)	3,823 (65.4%)		236 (55.3%)	258 (60.4%)	
Yes	229 (46.4%)	2,019 (34.6%)		191 (44.7%)	169 (39.6%)	
Chemotherapy			<0.001			0.933
No	93 (18.8%)	2,844 (48.7%)		90 (21.1%)	92 (21.5%)	
Yes	401 (81.2%)	2,998 (51.3%)		337 (78.9%)	335 (78.5%)	
Surgery^b^			<0.001			1.000
No	290 (58.7%)	4,201 (71.9%)		265 (62.1%)	264 (61.8%)	
Yes	204 (41.3%)	1,641 (28.1%)		162 (37.9%)	163 (38.2%)	
Subtype			<0.001			0.916
Luminal A	210 (42.5%)	3,213 (55.0%)		195 (45.7%)	195 (45.7%)	
Luminal B	125 (25.3%)	791 (13.5%)		105 (24.6%)	115 (26.9%)	
HER2-enriched	61 (12.3%)	413 (7.1%)		49 (11.5%)	43 (10.1%)	
Triple-negative	64 (13.0%)	701 (12.0%)		50 (11.7%)	48 (11.2%)	
Unknown	34 (6.9%)	724 (12.4%)		28 (6.6%)	26 (6.1%)	
Metastatic sites			0.019			0.076
Bone	172 (34.8%)	2,183 (37.4%)		159 (37.2%)	152 (35.6%)	
Brain	8 (1.6%)	82 (1.4%)		2 (0.5%)	6 (1.4%)	
Liver	49 (9.9%)	371 (6.4%)		34 (8.0%)	56 (13.1%)	
Lung	28 (5.7%)	480 (8.2%)		24 (5.6%)	18 (4.2%)	
Distant lymph nodes	39 (7.9%)	447 (7.7%)		30 (7.0%)	36 (8.4%)	
Multiple sites	198 (40.1%)	2,279 (39.0%)		178 (41.7%)	159 (37.2%)	

PSM, propensity score matching.

a Other races includes American Indians, Asians, and Pacific Islanders.

b Surgery only included surgery at the primary site.

### Relationship between age and metastasis patterns

**Figure 1:**
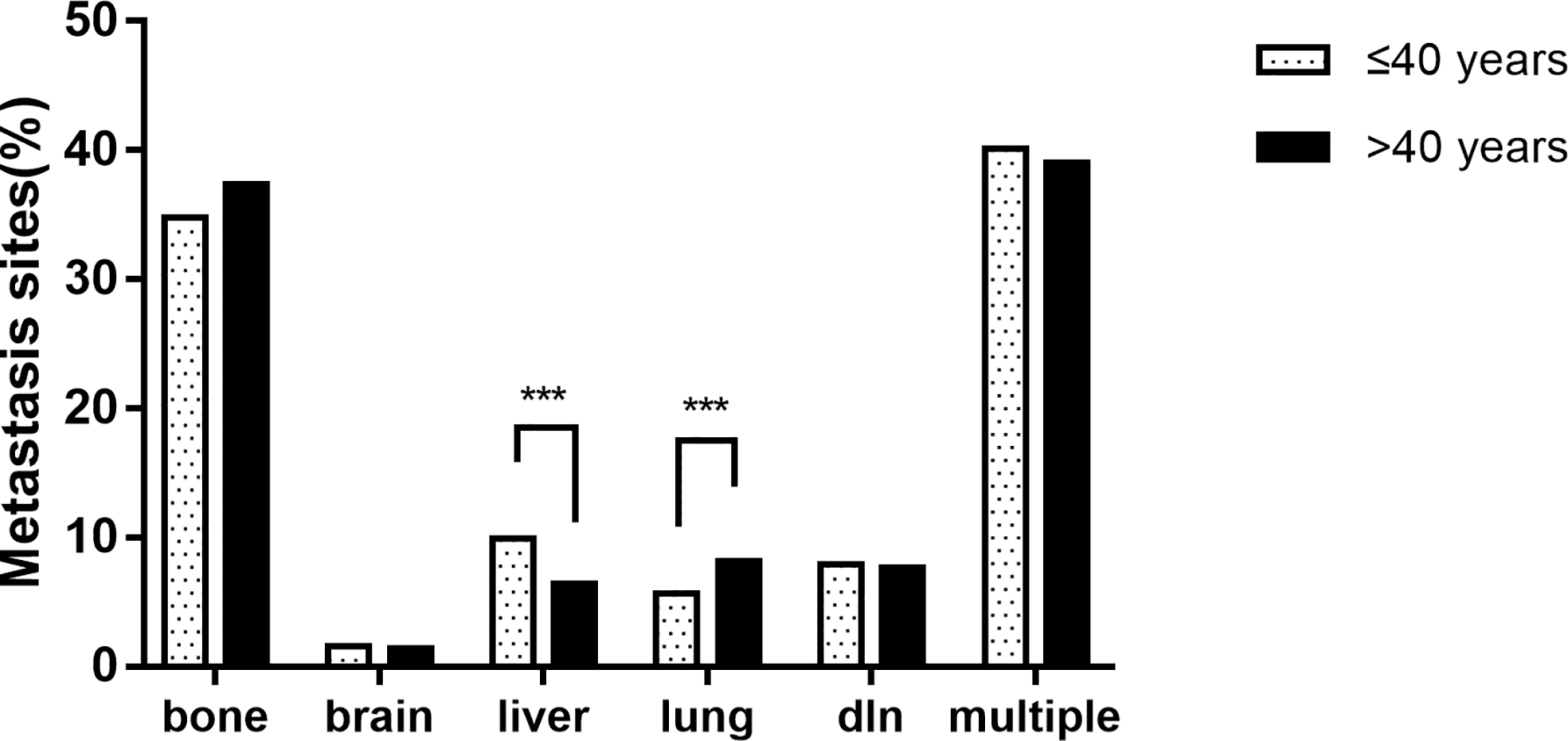
The proportion of metastatic sites of breast cancer patients between age groups (*** p < 0.001).

In the study population, bone was the most common site of metastasis (37.2%), followed by distant lymph node metastasis (7.7%), lung metastasis (8.0%), liver metastasis (6.6%), and brain metastasis (1.4%). The proportion of multiple sites metastasis was up to 39.1%, of which the most common was bone metastasis with liver metastasis, accounting for 16.1% of patients with multiple sites metastasis (n = 399). Notably, the proportion of liver-only metastasis was significantly higher in the younger group (9.9% vs. 6.4%, p = 0.002) than in the older group. The proportion of lung-only metastasis in the younger group (5.7% vs. 8.2%, p = 0.045) was significantly lower than that in the older group. There was no significant difference between the two groups in brain-only metastasis and distant lymph node-only metastasis. Detailed results are shown in **Figure 1**.

### Survival outcomes among age groups

**Figure 2:**
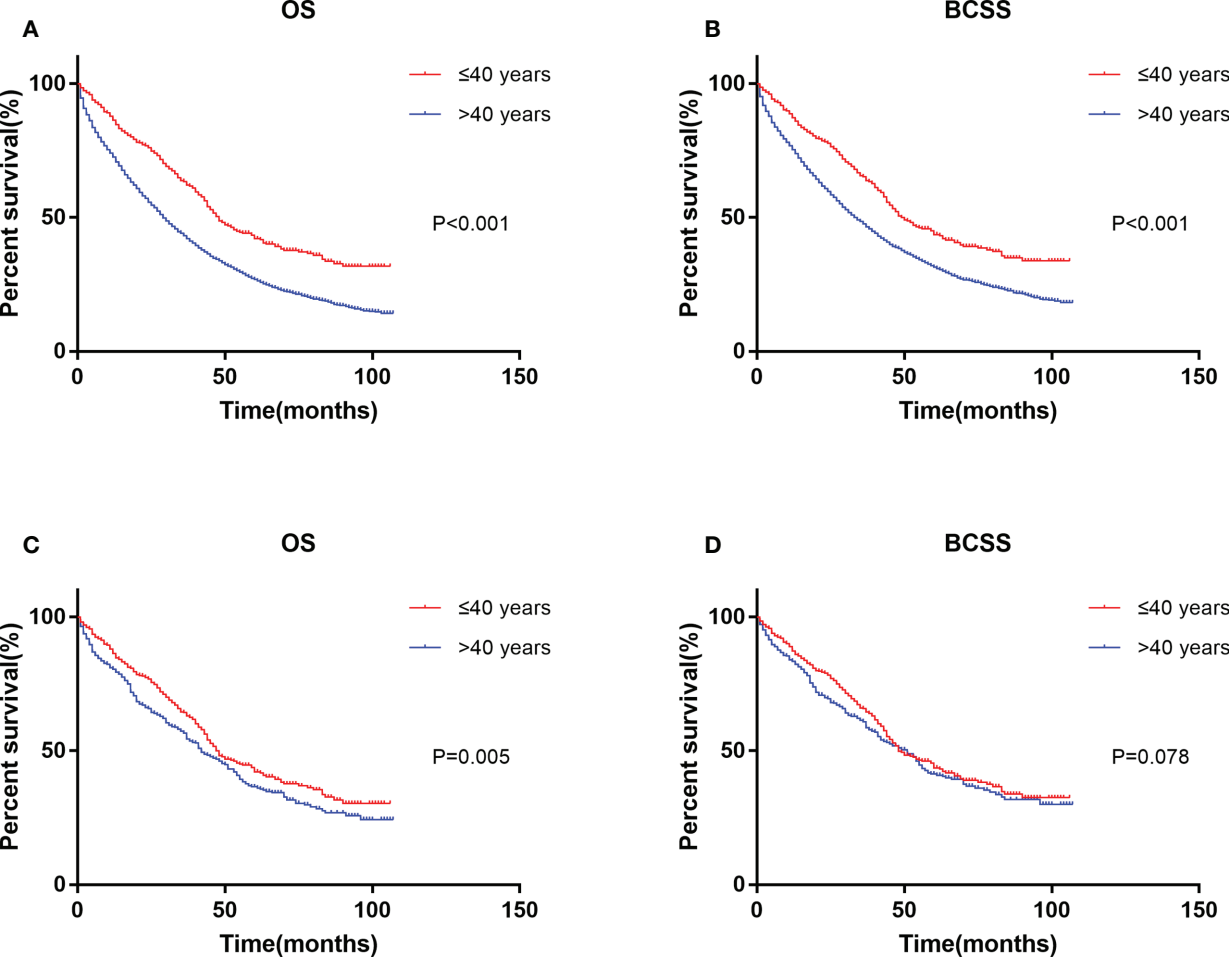
Kaplan–Meier curve of overall survival **(A)** and cancer-specific survival **(B)** by age groups before PSM. Kaplan–Meier curve of overall survival **(C)** and cancer-specific survival **(D)** after PSM. PSM, propensity score matching.

Kaplan–Meier analysis showed that the younger group had the best prognosis and the older group had the worst prognosis (**Figures 2A, B**). The median survival was 48 months in the younger group and 29 in the older group. The younger group had the best OS (HR: 0.599, 95% CI: 0.543–0.661, p < 0.001) and BCSS (HR: 0.645, 95% CI: 0.581–0.715, p < 0.001). Multivariate Cox regression showed that in addition to age, diagnosis year, race, T stage, N stage, subtype, surgery, chemotherapy, and metastasis sites were significantly correlated with survival (p < 0.05) (**Table 2**).

**Table 2:** Univariate and multivariate analyses of the overall survival (OS) and breast cancer-specific survival (BCSS) of the study population.

	Univariate analysis				Multivariate analysis			
Variable	BCSS		OS		BCSS		OS	
	HR (95% CI)	*p*	HR (95% CI)	*p*	HR (95% CI)	*p*	HR (95% CI)	*p*
Age								
≤40	1		1		1		1	
>40	1.560 (1.370–1.770)	<0.05	1.680 (1.490–1.900)	<0.05	1.402 (1.234–1.594)	<0.05	1.475 (0.678–1.303)	<0.05
Race								
White	1		1		1		1	
Black	1.270 (1.180–1.380)	<0.05	1.280 (1.190–1.380)	<0.05	1.231 (1.135–1.336)	<0.05	1.254 (0.797–1.162)	<0.05
Other/unknown	0.927 (0.830–1.040)	0.18	0.918 (0.827–1.020)	0.106	0.922 (0.825–1.029)	0.147	0.919 (1.088–0.828)	0.116
T stage								
T0/T1	1		1		1		1	
T2/T3	1.100 (1.000–1.220)	<0.05	1.090 (0.996–1.200)	0.06	1.183 (1.070–1.309)	<0.05	1.195 (0.837–1.087)	<0.05
T4	1.470 (1.330–1.630)	<0.05	1.460 (1.330–1.610)	<0.05	1.394 (1.253–1.550)	<0.05	1.426 (0.701–1.290)	<0.05
Tx	1.510 (1.350–1.690)	<0.05	1.530 (1.370–1.710)	<0.05	1.195 (1.059–1.348)	<0.05	1.199 (0.834–1.071)	<0.05
N stage								
N0	1		1		1		1	
N1/N2	0.990 (0.917–1.070)	0.806	0.936 (0.872–1.010)	0.072	1.028 (0.949–1.114)	0.504	0.978 (1.022–0.908)	0.568
N3	1.010 (0.916–1.120)	0.784	0.977 (0.888–1.080)	0.64	1.077 (0.966–1.202)	0.183	1.045 (0.957–0.943)	0.402
Nx	1.400 (1.250–1.560)	<0.05	1.400 (1.270–1.560)	<0.05	1.179 (1.047–1.327)	<0.05	1.186 (0.843–1.063)	<0.05
Radiation								
No	1		1		1		1	
Yes	0.927 (0.870–0.989)	<0.05	0.885 (0.833–0.941)	<0.05	1.076 (1.006–1.150)	<0.05	1.028 (0.973–0.965)	0.387
Chemotherapy								
No	1		1		1		1	
Yes	0.738 (0.694–0.785)	<0.05	0.693 (0.654–0.734)	<0.05	0.704 (0.655–0.756)	<0.05	0.669 (1.494–0.626)	<0.05
Surgery				<0.05				
No	1		1		1		1	
Yes	0.535 (0.498–0.575)	<0.05	0.537 (0.501–0.574)		0.574 (0.531–0.622)	<0.05	0.590 (1.695–0.548)	<0.05
Subtype								
Luminal A	1		1		1		1	
Luminal B	0.678 (0.613–0.750)	<0.05	0.670 (0.610–0.737)	<0.05	0.740 (0.666–0.823)	<0.05	0.751 (1.332–0.680)	<0.05
HER2-enriched	0.903 (0.796–1.024)	0.112	0.841 (0.744–0.949)	<0.05	1.079 (0.943–1.234)	0.268	1.037 (0.965–0.911)	<0.05
Triple-negative	2.568 (2.348–2.809)	<0.05	2.465 (2.263–2.685)	<0.05	3.238 (2.934–3.574)	<0.05	3.149 (0.318–2.867)	0.584
Unknown	1.538 (1.399–1.690)	<0.05	1.560 (1.428–1.704)	<0.05	1.411 (1.278–1.559)	<0.05	1.413 (0.708–1.287)	<0.05
Metastatic sites								<0.05
Bone	1		1		1		1	
Brain	2.470 (1.950–3.150)	<0.05	2.370 (1.880–2.980)	<0.05	2.111 (1.654–2.695)	<0.05	2.072 (0.483–1.642)	<0.05
Liver	1.090 (0.957–1.250)	0.188	1.080 (0.953–1.230)	0.228	1.320 (1.148–1.517)	<0.05	1.332 (0.751–1.169)	<0.05
Lung	1.170 (1.040–1.330)	<0.05	1.170 (1.050–1.320)	0.006	1.061 (0.935–1.204)	0.357	1.064 (0.940–0.946)	0.304
Distant lymph nodes	0.793 (0.691–0.909)	<0.05	0.820 (0.723–0.930)	0.002	0.863 (0.748–0.995)	<0.05	0.910 (1.099–0.798)	0.159
Multiple sites	1.690 (1.580–1.810)	<0.05	1.620 (1.520–1.730)	<0.05	1.634 (1.519–1.758)	<0.05	1.594 (0.628–1.488)	<0.05

OS, overall survival; BCSS, cancer-specific survival; HRs, hazard ratios; CI, confidence interval.

Since the uneven baseline characteristics may have a marked impact on the survival outcomes, we performed a 1:1 propensity score matching analysis to the utmost to eliminate the baseline variations. A total of 5,348 patients >40 years old were excluded due to a lack of definite baseline characteristics. Finally, 427 patients in the older group were selected to match 427 patients in the younger group. No significant differences were observed for all of the baseline variations between the matched groups (**Table 1**). After PSM, the younger group still exhibited a better clinical outcome than the older group (**Figures 2C, D**). Multivariate Cox regression after PSM showed that age, diagnosis year, race, T stage, N stage, subtype, surgery, chemotherapy, and metastasis sites were significantly correlated with OS (p < 0.05) (**Table 3**). However, age was not correlated with BCSS.

**Table 3:** Univariate and multivariate analyses of the OS and breast cancer-specific survival BCSS of the study population after PSM.

	Univariate analysis				Multivariate analysis			
Variable	BCSS		OS		BCSS		OS	
	HR (95% CI)	*p*	HR (95% CI)	*p*	HR (95% CI)	*p*	HR (95% CI)	*p*
Age								
18–39	1		1		1		1	
≥40	1.120 (0.929–1.340)	0.242	1.230 (1.030–1.460)	<0.05	1.205 (0.998–1.455)	0.052	1.324 (1.106–1.585)	<0.05
Race								
White	1		1		1		1	
Black	1.900 (1.540–2.330)	<0.05	1.840 (1.510–2.240)	<0.05	1.467 (1.184–1.819)	<0.05	1.411 (1.149–1.733)	<0.05
Other/unknown	1.270 (0.943–1.720)	0.116	1.230 (0.922–1.640)	0.159	1.159 (0.844–1.593)	0.362	1.100 (0.810–1.492)	0.542
T stage								
T0/T1	1		1		1		1	
T2/T3	1.080 (0.785–1.490)	0.637	0.996 (0.741–1.340)	0.977	1.193 (0.852–1.670)	0.305	1.133 (0.828–1.551)	0.435
T4	1.510 (1.080–2.130)	<0.05	1.410 (1.030–1.940)	<0.05	1.568 (1.084–2.2690	<0.05	1.507 (1.068–2.126)	<0.05
Tx	1.280 (0.859–1.910)	0.225	1.190 (0.816–1.720)	0.372	1.144 (0.755–1.732)	0.526	1.016 (0.688–1.501)	0.936
N stage								
N0	1		1		1		1	
N1/N2	0.839 (0.656–1.070)	0.161	0.806 (0.638–1.020)	0.071	0.800 (0.616–1.040)	<0.05	0.778 (0.606–0.999)	<0.05
N3	0.981 (0.720–1.340)	0.904	1.010 (0.755–1.350)	0.947	0.887 (0.635–1.241)	0.484	0.923 (0.673–1.267)	0.622
Nx	0.945 (0.596–1.500)	0.812	1.180 (0.7921–1.770)	0.41	0.762 (0.470–1.236)	0.271	1.005 (0.658–1.534)	0.983
Radiation								
No	1		1		1		1	
Yes	0.880 (0.731–1.060)	0.177	0.814 (0.682–0.973)	<0.05	1.154 (0.939–1.418)	0.175	1.051 (0.862–1.280)	0.624
Chemotherapy								
No	1		1		1		1	
Yes	0.684 (0.553–0.845)		0.667 (0.546–0.815)	<0.05	0.807 (0.634–1.028)	0.083	0.748 (0.595–0.939)	<0.05
Surgery								
No	1		1		1		1	
Yes	0.525 (0.430–0.642)	<0.05	0.550 (0.455–0.665)	<0.05	0.596 (0.474–0.748)	<0.05	0.632 (0.510–0.784)	<0.05
Subtype								
Luminal A	1		1		1		1	
Luminal B	0.508 (0.396–0.653)	<0.05	0.530 (0.419–0.671)	<0.05	0.503 (0.387–0.654)	<0.05	0.532 (0.415–0.682)	<0.05
HER2-enriched	0.541 (0.379–0.772)	<0.05	0.527 (0.373–0.744)	<0.05	0.560 (0.386–0.814)	<0.05	0.556 (0.387–0.798)	<0.05
Triple-negative	3.281 (2.517–4.276)	<0.05	3.407 (2.655–4.372)	<0.05	3.334 (2.451–4.534)	<0.05	3.375 (2.527–4.507)	<0.05
Unknown	1.402 (0.964–2.040)	0.077	1.412 (0.987–2.021)	0.059	1.290 (0.876–1.901)	0.198	1.317 (0.910–1.907)	0.145
Metastatic sites								
Bone	1		1		1		1	
Brain	10.100 (4.690–21.700)	<0.05	10.300 (5.010–21.100)	<0.05	10.537 (4.777–23.238)	<0.05	10.526 (5.024–22.052)	<0.05
Liver	0.936 (0.666–1.320)	0.702	0.996 (0.725–1.370)	0.982	1.083 (0.754–1.557)	0.666	1.120 (0.799–1.571)	0.511
Lung	0.890 (0.539–1.470)	0.647	1.000 (0.637–1.580)	0.992	0.722 (0.421–1.238)	0.236	0.826 (0.505–1.351)	0.446
Distant lymph nodes	0.581 (0.369–0.917)	<0.05	0.631 (0.414–0.960)	<0.05	0.650 (0.405–1.045)	0.075	0.661 (0.427–1.025)	0.065
Multiple sites	1.700 (1.380–2.080)	<0.05	1.660 (1.360–2.020)	<0.05	1.459 (0.685–1.837)	0.001	1.429 (1.146–1.783)	<0.05

OS, overall survival; BCSS, cancer-specific survival; HRs, hazard ratios; CI, confidence interval.

**Figure 3:**
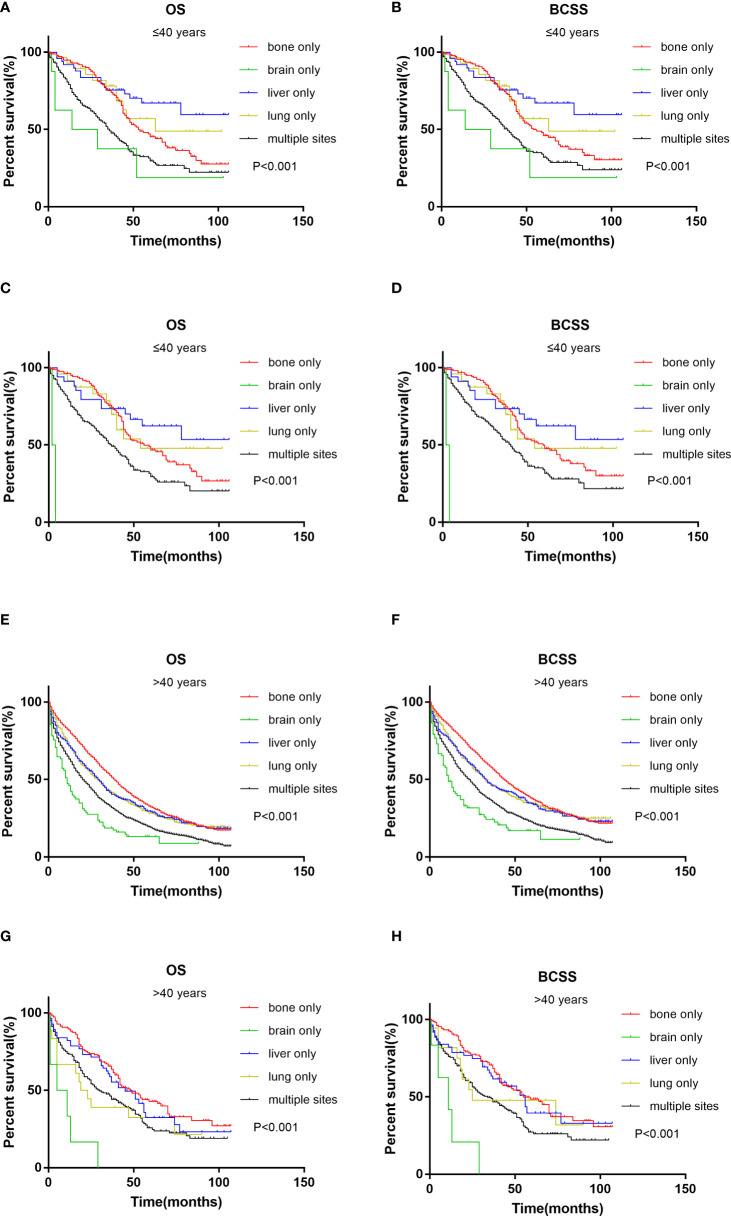
Kaplan–Meier curve of overall survival and cancer-specific survival according to metastasis sites in younger (≤40 years) groups before **(A, B)** and after **(C, D)** PSM; Kaplan–Meier curve of overall survival and cancer-specific survival according to metastasis sites in older (>40 years) groups before **(E, F)** and after **(G, H)** PSM.

The survival analysis of metastatic sites in different age groups showed that in the younger group, patients with liver only metastasis had the best OS and BCSS (**Figures 3A, B**). The results were the same after PSM (**Figures 3C, D**). However, in the older group, patients with bone only metastasis had the best OS and BCSS (**Figures 3E, F**), also after PSM (**Figures 3G, H**). Patients with brain only metastasis had the worst prognosis in both age groups.

**Figure 4:**
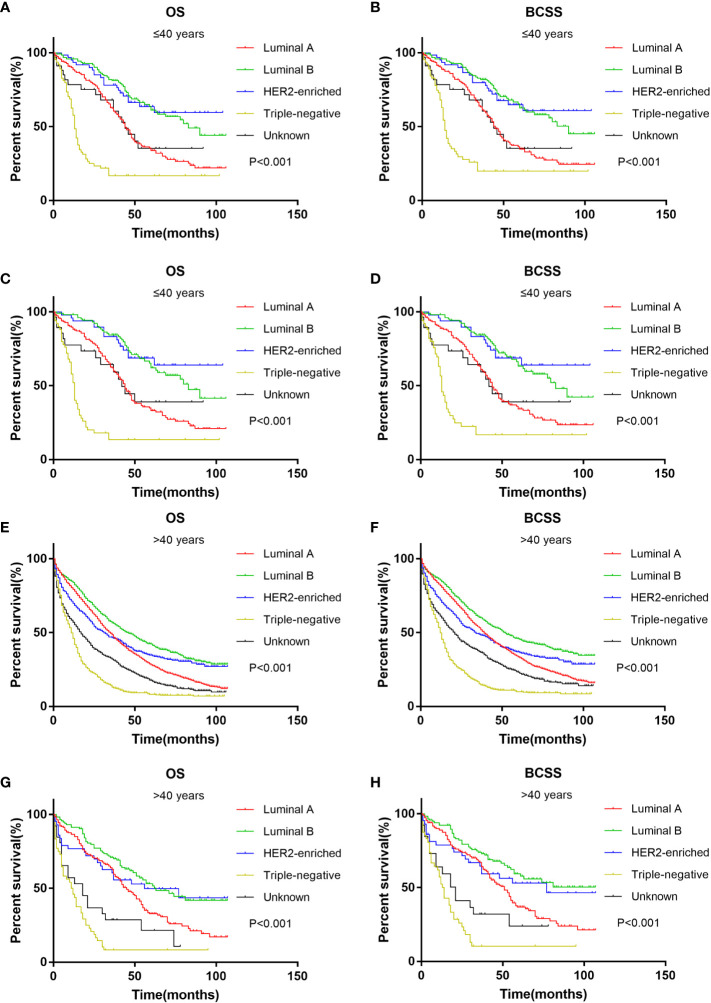
Kaplan-Meier curve of overall survival and cancer-specific survival according to BC subtype in younger (≤40 years) groups before **(A, B)** and after **(C, D)** PSM; Kaplan-Meier curve of overall survival and cancer-specific survival according to BC subtype in older (>40 years) groups before **(E, F)** and after **(G, H)** PSM.

Survival analysis of different age groups showed that HER2-enriched subtype and Luminal B subtype had the best prognosis in both age groups (**Figures 4A, B, E, F**). The results were the same after PSM (**Figures 4C, D, G, H**). Triple-negative subtype had the worst prognosis in both age groups.

## Discussion

The total number of young breast cancer is relatively small, but the incidence of distant metastasis is increasing. The incidence of MBC among young women increased from 1.53 per 100,000 to 2.90 per 100,000 from 1976 to 2009, with the trend showing no slowing down, indicating increasing epidemiological and clinical significance (5). However, there are few studies on young women with MBC. Our study divided MBC patients into two age groups in a large population-based SEER data to evaluate the effect of age on metastasis patterns and the effect of age, metastasis sites, and BC subtype on prognosis.

Our study showed that young MBC patients had larger tumors, higher rates of lymph node involvement, and more aggressive BC subtypes, consistent with previous studies. The largest study to date involving 200,000 breast cancer patients was conducted by Gnerlichetal with 15,000 breast cancer patients aged <40 years at diagnosis, finding that younger women were more likely to be diagnosed with larger tumors, lymph node involvement, poorly differentiated tumors, and estrogen receptor(ER)-tumors (16). In addition, a California Cancer Registry study including 5,600 women aged <40 years at diagnosis reported a statistically significant HER2-higher expression in younger women (17). The results of these studies demonstrate the aggressiveness of breast cancer in young patients. In addition, screening is poor in young women due to the low incidence of breast cancer, which leads to more severe disease at the time of diagnosis (13).

The metastasis patterns in different age groups remain controversial. A study of 14,403 patients based on the Epidemiological Strategy and Medical Economics (ESME) database found that MBC patients aged <40 years were more likely to have visceral metastasis than bone metastasis (12). Chen et al. found that MBC patients aged <50 years were more likely to have distant lymph node metastasis and multiple sites of metastasis and less likely to have lung metastasis (18). A study of 6,640 patients showed that MBC patients aged <40 years were more likely to have brain and liver metastases than patients aged ≥40 years (19). We found that MBC patients aged ≤40 years had a higher risk of liver metastasis, while their risk of lung metastasis was lower. This is consistent with the study of R. Ogiya, in which young women with Luminal A MBC have shown a higher incidence of liver metastases at diagnosis as compared to older women, who are more frequently diagnosed with bone-only disease (20). This may be due to more HER2+ and triple-negative subtypes in young patients. Previous studies have shown that the HER2+ subtype is associated with liver metastasis (21, 22). HER2+ and triple-negative subtypes were more likely to have visceral metastasis than bone metastasis (23). However, the underlying molecular mechanisms need further research.

Multivariate Cox regression showed that the OS of the younger group was better than that of the older group, and youth was an independent protective factor of OS. However, the result is not shown in BCSS. This is contrary to previous studies that reported that young age at diagnosis is associated with poor prognosis in breast cancer. There are many reasons for this. First, our study confirmed that younger patients have fewer comorbidities (24), while older patients have decreased physiologic function and an increased risk of non-cancer death. Younger patients were more likely to receive treatment (surgery and chemotherapy), consistent with previous studies (18). In addition to conventional systemic treatment, several studies have suggested that MBC patients also benefit from local treatment (25, 26). Second, there were more HER2+ subtypes in young patients. Studies have found that the improvement of OS in MBC patients is mainly driven by the HER2+ subgroup (27). In fact, new HER2-targeted therapies were released in 2013 (namely, pertuzumab and T-DM1), which were associated with major OS benefits in clinical trials (28–30). In a real-world study of patients with HER2+ MBC, younger patients were more likely to receive PH+taxane than older patients, and older patients were more likely to receive regimens with H without P or hormone therapy. It turned out younger patients have better BCSS than older patients (31). The ESME observational study showed that chemotherapy and anti-HER2 therapies were less frequently used in older patients, resulting in shorter OS in women >60 years (32). This may also explain why our study found that HER2-enriched subtypes had the best prognosis in the younger group. This suggests that there may be undertreatment in elderly patients to some extent.

Kaplan–Meier analysis showed interesting results. In the younger group, patients with liver-only metastasis, not bone-only metastasis, had the best prognosis. This may be due to the fact that 59.2% of patients with liver metastasis were HER2+ subtypes in the younger group of our study, and younger patients received more chemotherapy and anti-HER2 therapy than older patients, bringing survival benefits. S. Sakhuja et al. (33) found that HER2-enriched and Luminal B subtypes had the best survival in patients with liver metastasis. Ji et al. (21) also reached the same conclusion. Similarly, the HER2+ subtype in the younger group also showed a survival advantage in our study. This suggests that even in metastatic HER2+ breast cancer, anti-HER2 therapy also results in considerable and long-lasting improvements in survival (34). Moreover, there may also be bias in patient selection, and the specific mechanism needs to be further explored.

There are some limitations to this study. As a prospective study, inherent selection biases cannot be avoided and could limit the external validity of this study. The SEER database does not provide information on targeted therapy or endocrine therapy, which may affect survival outcomes. In addition, the SEER database only provided HER2 information after 2010, resulting in the insufficient follow-up of some patients. Despite these limitations, this study elucidates the metastasis patterns and prognostic characteristics based on metastasis sites and BC subtypes in young MBC patients. These findings may provide a basis for the precision treatment of young MBC patients.

## Data availability statement

The datasets presented in this study can be found in online repositories. The names of the repository/repositories and accession number(s) can be found in the article/supplementary material.

## Author contributions

WZ, JL, and YL designed the study. SW, XM, XZ, CY, MC, and SZ extracted and analyzed the data. WZ and JL wrote and edited the manuscript. The authors were ranked according to their contributions. All authors contributed to the article and approved the submitted version.

## Conflict of interest

The authors declare that the research was conducted in the absence of any commercial or financial relationships that could be construed as a potential conflict of interest.

## Publisher’s note

All claims expressed in this article are solely those of the authors and do not necessarily represent those of their affiliated organizations, or those of the publisher, the editors and the reviewers. Any product that may be evaluated in this article, or claim that may be made by its manufacturer, is not guaranteed or endorsed by the publisher.
